# 

**Published:** 1903-11-07

**Authors:** 


					THE HOSPITAL. "The Standard of highest purity."?Lancet. November 7th, 1903
CADBURY'S ^ U ]& CADBURY'S
COCOA
'A PERFECT FOOD."
GOCOA
NO DRUGS OR ALKALIB8 UIID.
No. 893] Vol, XXXV. Saturday,
Nov. 7th, 1903.
[SmbacrlptUn^TB!ms,] pR|(;E THREEPEHCE.

				

